# ALKBH1 deficiency leads to loss of homeostasis in human diploid somatic cells

**DOI:** 10.1007/s13238-020-00744-4

**Published:** 2020-07-13

**Authors:** Hongyu Li, Zeming Wu, Xiaoqian Liu, Sheng Zhang, Qianzhao Ji, Xiaoyu Jiang, Zunpeng Liu, Si Wang, Jing Qu, Weiqi Zhang, Moshi Song, Eli Song, Guang-Hui Liu

**Affiliations:** 1 National Laboratory of Biomacromolecules, CAS Center for Excellence in Biomacromolecules, Institute of Biophysics, Chinese Academy of Sciences, Beijing, 100101 China; 2 State Key Laboratory of Stem Cell and Reproductive Biology, Institute of Zoology, Chinese Academy of Sciences, Beijing, 100101 China; 3 State Key Laboratory of Membrane Biology, Institute of Zoology, Chinese Academy of Sciences, Beijing, 100101 China; 4 Institute for Stem Cell and Regeneration, Chinese Academy of Sciences, Beijing, 100101 China; 5 University of Chinese Academy of Sciences, Beijing, 100049 China; 6 Advanced Innovation Center for Human Brain Protection, National Clinical Research Center for Geriatric Disorders, Xuanwu Hospital Capital Medical University, Beijing, 100053 China

**Dear Editor,**

As the most prevalent DNA methylation modification in prokaryotes, DNA N6-methyladenosine (6mA) in eukaryotic genomes has recently been observed in diverse species including *Caenorhabditis elegans* (Greer et al., 2015), *Drosophila melanogaster* (Zhang et al., 2015), mouse (Wu et al., 2016) and human (Xiao et al., 2018). 6mA has been reported to associate with multiple physiological processes including embryonic development and tumorigenesis (Greer et al., 2015; Zhang et al., 2015; Xie et al., 2018), yet some controversies exist. In contrast to the findings showing that ALKBH1 (alkB homolog 1) is a primary 6mA demethylase in mouse and human cells (Wu et al., 2016; Xiao et al., 2018; Xie et al., 2018), other studies indicate that ALKBH1 is prone to demethylate 6mA on bubbled or bulged DNAs that are often featured by a locally unpairing region with flanking duplex, such as D-loop, R-loop as well as DNA or RNA stem-loop, and single-stranded DNAs at a lower efficiency, but not double-stranded DNAs (Tian et al., 2020; Zhang et al., 2020a). Stem cell exhaustion is a major causal and risk factor underlying the progressive disruption of physiological integrity during the development of aging-associated disorders, in which epigenetic alterations are closely implicated (Zhang et al., 2020b). Yet, the roles of 6mA and its putative regulators such as ALKBH1 in the homeostatic maintenance of human stem cells and their differentiated derivatives remain elusive.

To investigate the role of ALKBH1 in regulating homeostatic maintenance in human diploid cells, we first generated ALKBH1-deficient human embryonic stem cells (hESCs) via clustered regularly interspaced short palindromic repeat/CRISPR-associated protein 9 (CRISPR/Cas9)-mediated non-homologous end joining (NHEJ) (Fig. 1A, 1B and S1A). The absence of the ALKBH1 protein was verified by western blotting (Fig. 1C). Phenotypic analyses revealed that *ALKBH1*^−/−^ hESCs expressed pluripotency markers including NANOG, SOX2 and OCT4 (Fig. 1D) and maintained a normal karyotype (Fig. 1E). Altogether, these data suggest that *ALKBH1*^−/−^ hESCs maintain normal pluripotency.

**Figure 1:**
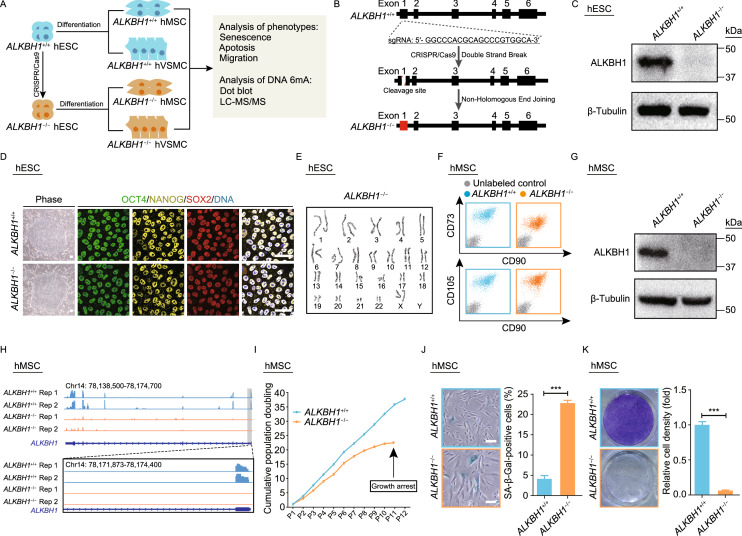

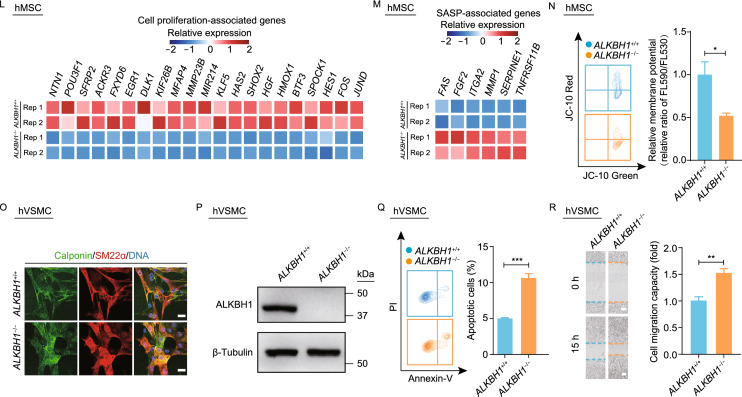
**Generation and phenotypic analyses of** ***ALKBH1***^**−/−**^ **hESCs, hMSCs and hVSMCs**. (A) Schematic diagram of the generation of ALKBH1-deficient hESCs and derived hMSCs and hVSMCs. (B) Illustration of *ALKBH1*-targeting strategy by CRISPR/Cas9-mediated non-homologous end-joining (NHEJ). (C) Western blotting analysis of ALKBH1 in *ALKBH1*^+/+^ and *ALKBH1*^−/−^ hESCs. β-Tubulin was used as a loading control. (D) Immunostaining of pluripotency markers including NANOG, SOX2 and OCT4 in *ALKBH1*^+/+^ and *ALKBH1*^−/−^ hESCs. Scale bar, 50 μm. (E) Karyotyping analysis of *ALKBH1*^−/−^ hESCs. (F) FACS analysis of the MSC-specific surface markers CD73, CD90 and CD105 in *ALKBH1*^+/+^ and *ALKBH1*^−/−^ hMSCs. (G) Western blotting analysis of ALKBH1 in *ALKBH1*^+/+^ and *ALKBH1*^−/−^ hMSCs. β-Tubulin was used as a loading control. (H) Top, representative track showing the mRNA abundance of ALKBH1 in *ALKBH1*^+/+^ and *ALKBH1*^−/−^ hMSCs. Bottom, sgRNA targeting site for *ALKBH1* (exon 1) and its flanking region are zoomed. (I) Growth curve analysis of *ALKBH1*^+/+^ and *ALKBH1*^−/−^ hMSCs. Data are presented as the mean ± SEM. *n* = 3. (J) SA-β-Gal staining of *ALKBH1*^+/+^ and *ALKBH1*^−/−^ hMSCs at passage 10. Scale bar, 100 μm. Data are presented as the mean ± SEM. *n* = 3. ***, *P* < 0.001. (K) Clonal expansion analysis of *ALKBH1*^+/+^ and *ALKBH1*^−/−^ hMSCs at passage 10. Data are presented as the mean ± SEM. *n* = 3. ***, *P* < 0.001. (L) Heatmap showing the relative expression levels of genes associated with cell proliferation in *ALKBH1*^+/+^ and *ALKBH1*^−/−^ hMSCs. (M) Heatmap showing the relative expression levels of genes associated with SASP in *ALKBH1*^+/+^ and *ALKBH1*^−/−^ hMSCs. (N) FACS analysis of the JC-10 staining in *ALKBH1*^+/+^ and *ALKBH1*^*−/−*^ hMSCs at passage 3. Data are presented as the mean ± SEM. *n* = 3. *, *P* < 0.05. (O) Immunostaining of VSMC-specific markers Calponin and SM22α in *ALKBH1*^+/+^ and *ALKBH1*^−/−^ hVSMCs. Scale bar, 25 μm. (P) Western blotting analysis of ALKBH1 in *ALKBH1*^+/+^ and *ALKBH1*^−/−^ hVSMCs. β-Tubulin was used as a loading control. (Q) FACS analysis of apoptotic cells by Annexin-V and propidium iodide (PI) labeling in *ALKBH1*^+/+^ and *ALKBH1*^−/−^ hVSMCs. Data are presented as the mean ± SEM. *n* = 3. ***, *P* < 0.001. (R) Wound healing assay for the analysis of migration ability of *ALKBH1*^+/+^ and *ALKBH1*^−/−^ hVSMCs. Scale bar, 100 μm. Data are presented as the mean ± SEM. *n* = 4. *, *P* < 0.05; **, *P* < 0.01

We next differentiated *ALKBH1*^+/+^ and *ALKBH1*^−/−^ hESCs into human mesenchymal stem cells (hMSCs) and vascular smooth muscle cells (hVSMCs) (Fig. 1A). *ALKBH1*^−/−^ hMSCs expressed MSC-specific surface markers including CD73, CD90 and CD105 (Fig. 1F), and demonstrated a lack of ALKBH1 expression (Fig. 1G, 1H and S1B). Whole-genome sequencing (WGS) showed that ALKBH1 deficiency did not impair the genomic integrity in hMSCs (Fig. S1C). Early-onset growth arrest of *ALKBH1*^−/−^ hMSCs was observed through serial passaging, relative to that of *ALKBH1*^+/+^ hMSCs (Fig. 1I). Besides, *ALKBH1*^−/−^ hMSCs exhibited higher percentage of senescence-associated β-galactosidase (SA-β-Gal)-positive cells and lower clonal expansion ability (Fig. 1J and 1K). Genome-wide RNA sequencing (RNA-seq) showed that ALKBH1 deficiency resulted in downregulation of some master genes promoting cell proliferation (e.g., *FOS*, *JUND*, *HMOX1*) and upregulation of senescence-associated secretory phenotype (SASP) genes (e.g., *MMP1* and *SERPINE1*) (Figs. S1D, 1L and 1M). We did not observe any significant increase in γH2AX and 53BP1 foci in the nuclei of *ALKBH1*^−/−^ hMSCs, indicative of no increase in DNA damage response upon ALKBH1 depletion (Fig. S1E). Consistent with the previous report that ALKBH1 deficiency causes mitochondrial damage in HEK293T cells (Kawarada et al., 2017), we observed mitochondrial depolarization in *ALKBH1*^−/−^ hMSCs (Fig. 1N). These data indicate that ALKBH1 deficiency accelerates hMSC senescence. On the other hand, *ALKBH1*^+/+^ and *ALKBH1*^−/−^ hVSMCs had comparable expression levels of VSMC-specific markers such as Calponin and SM22α (Fig. 1O). The absence of ALKBH1 protein expression in *ALKBH1*^−/−^ hVSMCs was confirmed by Western blotting (Fig. 1P). Phenotypically, increased apoptosis and enhanced migration ability were observed in *ALKBH1*^−/−^ hVSMCs, compared with those in *ALKBH1*^+/+^ hVSMCs (Fig. 1Q and 1R). Altogether, these results indicate that ALKBH1 is required to maintain the homeostasis of hMSCs and hVSMCs, and deficiency of ALKBH1 results in mitochondrial depolarization and premature senescence in hMSCs and increased apoptosis in hVSMCs.

We further investigated whether the abnormalities observed in *ALKBH1*^−/−^ hMSCs and hVSMCs were associated with changes in 6mA levels. Dot blot assay analysis showed comparable 6mA levels between *ALKBH1*^+/+^ and *ALKBH1*^−/−^ hMSCs (Fig. 2A). Furthermore, liquid chromatography coupled with tandem mass spectrometry (LC-MS/MS) analysis revealed no alteration in 6mA levels between *ALKBH1*^+/+^ and *ALKBH1*^−/−^ hESCs, between *ALKBH1*^+/+^ and *ALKBH1*^−/−^ hMSCs, and between *ALKBH1*^+/+^ and *ALKBH1*^−/−^ hVSMCs, respectively (Fig. 2B–E and S1F). The absence of mycoplasma contamination in cultured cells was demonstrated by PCR analysis (Fig. S1G). WGS analysis further validated no contamination of the mycoplasma (*Mycoplasma genitalium* and *Mycoplasma hyorhinis*), bacterial (*Staphylococcus aureus* and *Escherichia coli*) or fungi (*Aspergillus fumigatus*) genomic DNA in the genomes of *ALKBH1*^+/+^ and *ALKBH1*^−/−^ hMSCs (Fig. 2F). To test whether the 6mA signals detected could be from the contamination by the reagents (e.g., enzymes and buffers) used in LC-MS/MS assay, LC-MS/MS was performed with a negative control (ddH_2_O instead of gDNA) and only very low signal was detected (Fig. 2G). Altogether, these data demonstrate that depletion of ALKBH1 exerts minimal impact on 6mA levels in hESCs and their differentiated derivatives and that ALKBH1 regulates the homeostasis of hMSCs and hVSMCs possibly in a DNA 6mA-independent manner.

**Figure 2:**
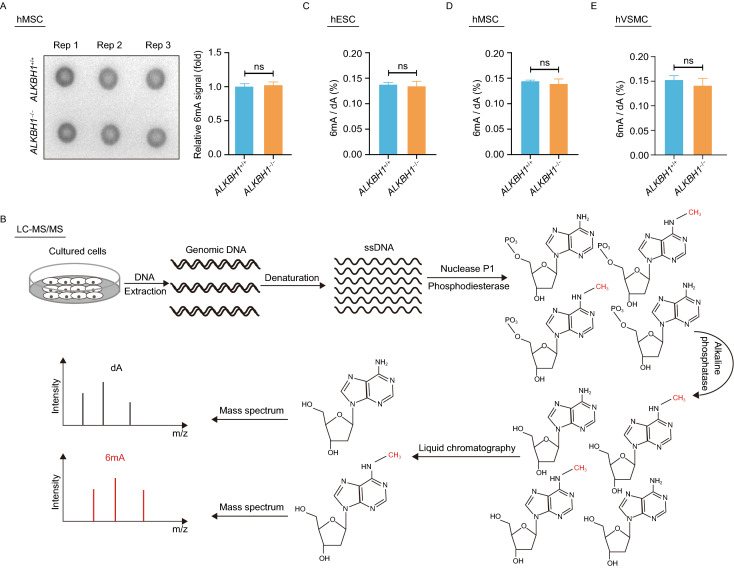

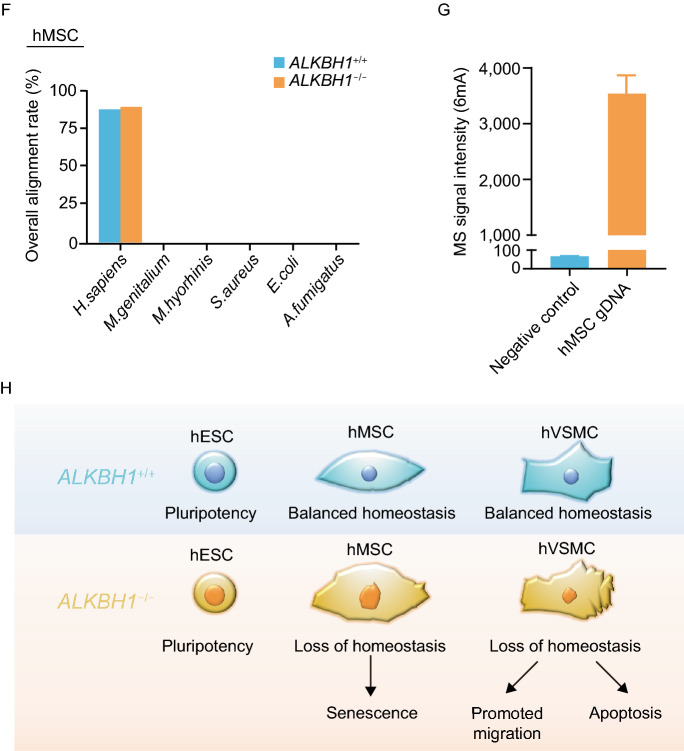
**Detection of 6mA levels in** ***ALKBH1***^**−/−**^ **hESCs, hMSCs and hVSMCs**. (A) Dot blotting showing 6mA in *ALKBH1*^+/+^ and *ALKBH1*^−/−^ hMSCs. Data are presented as the mean ± SEM. *n* = 3. ns, not significant. (B) Schematic representation of quantification of 6mA in gDNA by LC-MS/MS. (C–E) Quantification of 6mA in gDNA by LC-MS/MS in *ALKBH1*^+/+^ and *ALKBH1*^−/−^ hESCs (C), hMSCs (D) and hVSMCs (E). Data are presented as the mean ± SEM. *n* = 3. ns, not significant. (F) Whole genome analysis of mycoplasma, bacteria and fungi contaminations in *ALKBH1*^+/+^ and *ALKBH1*^−/−^ hMSCs. Mycoplasma is represented by *Mycoplasma genitalium* (*M*. *genitalium*) and *Mycoplasma hyorhinis* (*M*. *hyorhinis*). Bacteria is represented by *Staphylococcus aureus* (*S. aureus*) and *Escherichia coli* (*E*. *coli)*. Fungi is represented by *Aspergillus fumigatus* (*A*. *fumigatus*). (G) Quantification of 6mA in gDNA and a negative control (ddH_2_O instead of gDNA) by LC-MS/MS. (H) Schematic graph showing the major effects of ALKBH1 deficiency on the homeostatic maintenance in hESCs, hMSCs and hVSMCs

In this study, we generated ALKBH1-knockout hESCs by using CRISPR/Cas9-based gene editing and further obtained their hMSC and hVSMC derivatives via directed differentiation. Overall, DNA 6mA levels were unaffected by ALKBH1 depletion in hESCs, hMSCs and hVSMCs. While ALKBH1 was dispensable for the maintenance of hESC pluripotency, ALKBH1-deficient hMSCs exhibited mitochondrial depolarization and early-onset senescence phenotypes, and loss of ALKBH1 increased apoptosis and migration ability of hVSMCs (Fig. 2H). All these cellular events appear to act independently of cellular 6mA levels.

Our data, together with previous studies, support the notion that ALKBH1 may play cell type- or species-specific roles. ALKBH1 did not adversely affect hESC pluripotency as evidenced by the normal expression of pluripotency markers in *ALKBH1*^−/−^ hESCs and their successful differentiation into hMSCs and hVSMCs. In a mouse-based study, ALKBH1 deficiency delays mouse embryonic stem cell (mESC) differentiation and increases apoptosis in differentiated mouse neural progenitor cells (mNPCs) (Ougland et al., 2012). Our data reveal that ALKBH1 deficiency accelerates hMSC senescence and increases apoptosis and migration ability in hVSMCs. Therefore, ALKBH1 appears to be vital for the homeostatic maintenance of human diploid adult stem cells (e.g., hMSCs) and terminally differentiated cells (e.g., hVSMCs). The biological function of ALKBH1 in other types of hESC derivatives awaits further investigations.

Despite that ALKBH1 has recently been reported as a 6mA demethylase (Xiao et al., 2018; Xie et al., 2018), which is by far the only one identified in human, we did not observe discernible difference in the 6mA levels in hESCs, hMSCs or hVSMCs caused by ALKBH1 deficiency, consistent with the recent report that ALKBH1 fails to eliminate 6mA in mESCs or HEK293T cells (Liu et al., 2020). Given that two studies have shown that ALKBH1 preferably demethylates 6mA on unpaired DNAs (Tian et al., 2020; Zhang et al., 2020a), our results imply that there might be few unpaired DNAs in the normally cultured hESCs, hMSCs and hVSMCs. For example, R-loop, a form of unpaired DNAs, is a known ALKBH1 substrate that only accounts for about 5% of the human genome (Sanz et al., 2016; Zhang et al., 2020a). Accordingly, the potential alteration in 6mA levels on R-loop may be undetectable in our study. Whether ALKBH1 deficiency affects the 6mA levels on the unpaired DNAs and contributes to the phenotypic abnormalities observed in hMSCs and hVSMCs warrants further investigations. It should be noted that several other studies have suggested ALKBH1 as a primary 6mA demethylase (Wu et al., 2016; Xiao et al., 2018; Xie et al., 2018). This divergence may stem from the cell-type specificity as the cells employed in those studies are mostly human HEK293T cells and transformed human cell lines, which harbor different characteristics compared to human diploid stem cells and their derivatives tested in this study. In addition, ALKBH1 has been implicated in the demethylation of N1-methyladenosine (m1A) on cytoplasmic tRNAs, thus affecting translation initiation and elongation (Liu et al., 2016). ALKBH1 was also reported to mediate the formation of 5-formylcytosine (f5C) from 5-methylcytosine (m5C) on mitochondrial tRNA^Met^ in HEK293T cells (Kawarada et al., 2017). Similar to the report that ALKBH1-deficient HEK293T cells exhibit mitochondrial damage (Kawarada et al., 2017), we detected a decrease in mitochondrial membrane potential in *ALKBH1*^−/−^ hMSCs. Therefore, our observations on the phenotypic defects of ALKBH1-deficient hMSCs and hVSMCs such as accelerated senescence, increased apoptosis and migration, may depend on its activity on mitochondrial regulation. It is also possible that some other proteins might be functionally redundant to ALKBH1 and mediate DNA 6mA demethylation, thus compensating for the effects caused of ALKBH1 deficiency. For instance, hALKBH5 has been reported to possess 6mA demethylation activity *in vitro* (Tian et al., 2020).

It should be emphasized that our current understanding of 6mA is relatively elementary. Recent studies have demonstrated that 6mA is incorporated into the mammalian genomic DNA in the process of DNA replication by DNA polymerase in a 6mA methylase-independent manner (Liu et al., 2020; Musheev et al., 2020). Another study reported 6mA as merely a false-positive signal likely caused by mycoplasma contamination and/or nonspecific antibodies (Douvlataniotis et al., 2020). While we and others performed experiments to exclude the possibilities of 6mA contaminations from cell culture or from the reagents used in LC-MS/MS assay (Liu et al., 2020), proving the existence and primary origin of human genomic DNA 6mA is still a challenge in this field. With full consideration of these caveats, our study indicates that ALKBH1 is a key homeostatic regulator of human diploid adult stem cells and terminally differentiated cells. These new findings undoubtedly add a new layer of biological complexity of ALKBH1 and its related pathways in the regulation of basic cellular physiology.

## Electronic supplementary material

Below is the link to the electronic supplementary material.

Electronic supplementary material 1 (PDF 719 kb)
