# Supplementary material for: ALKBH1 deficiency leads to loss of homeostasis in human diploid somatic cells
Source: Protein Cell. 2020 Jul 13;11(9):688–95. doi: 10.1007/s13238-020-00744-4 (PMC7452965; doi:10.1007/s13238-020-00744-4)
Supplement: Supplementary file 1 — Electronic supplementary material 1 (PDF 719 kb) [file 13238_2020_744_MOESM1_ESM.pdf]

Materials and Methods

Cell culture

ALKBH1$^{+/+}$ hESCs (human H9 ESCs, WiCell Research) and *ALKBH1*$^{-/-}$ hESCs were maintained on mitomycin C (Selleck)-inactivated mouse embryonic fibroblast (MEF) feeder in hESC culture medium that contains 80% DMEM/F12 (Gibco) medium, 20% Knockout Serum Replacement (Gibco), 0.1 mM non-essential amino acids (NEAA, Gibco), 2 mM GlutaMAX (Gibco), 1% penicillin/streptomycin (Gibco), 55 μM β-Mercaptoethanol (Thermo Fisher Scientific) and 10 ng/mL bFGF (Joint Protein Central, JPC). hESCs were cultured in mTeSR medium (STEMCELL Technologies) on Matrigel (Corning)-coated plates (Corning). hMSCs were cultured in hMSC culture medium containing 90% α-MEM medium with GlutaMAX (Gibco), 10% fetal bovine serum (FBS, Gibco), 0.1 mM NEAA (Gibco), 1% penicillin/streptomycin (Gibco) and 1 ng/mL bFGF (JPC) on Gelatin (Sigma)-coated plates. hVSMCs were cultured in hVSMC culture medium containing 50% DMEM/F12 (Gibco), 50% Neurobasal medium (Gibco), 1 × B27 supplement without Vitamin A (Gibco), 1 × N2 supplement (Gibco), 1% penicillin/streptomycin (Gibco), 55 μM β-Mercaptoethanol (Thermo Fisher Scientific) and 10 ng/mL recombinant Human PDGF-AB (PeproTech) on Gelatin-coated plates.

Generation of ALKBH1-knockout hESCs

ALKBH1$^{-/-}$ hESCs were generated by CRISPR/Cas9-mediated gene-editing technique as described previously (Cheng et al., 2019). In brief, single guide RNA (sgRNA) targeting the exon 1 of *ALKBH1* gene was designed and cloned into sgRNA vector (Addgene, #41824). Before electroporation, hESCs were treated with ROCK inhibitor Y-27632 (Selleck) for 8 hr and dissociated into single cells with TrypLE Express (Gibco). 5 × 10^6 hESCs were electroporated with 7 μg pCAGmCherry-gRNA (Addgene, #87110) vectors and 14 μg pCAG-1BPNLS-Cas9-1BPNLS-2AGFP (Addgene, #87109) vectors. hESCs were cultured on Matrigel-coated plates treated with ROCK inhibitor Y-27632. After 48 hr, mCherry/GFP-double positive hESCs were sorted by flow cytometer (BD, Aria II) and cultured on mitomycin C-inactivated MEFs in hESC culture medium. The emerging colonies were transferred onto Matrigel-coated 24-well plates (Corning) and expanded for subsequent analyses.
The sequence of *ALKBH1* sgRNA is 5′-GGCCCACGCAGCCCGTGGCA-3′.

Generation of hMSCs

The differentiation of *ALKBH1*$^{+/+}$ and *ALKBH1*$^{-/-}$ hMSCs was performed as described previously (Cheng et al., 2019; Deng et al., 2019). In brief, hESCs were dissociated into embryonic body and cultured on Matrigel-coated plates in hMSC differentiation medium containing 90% α-MEM medium with GlutaMAX (Gibco), 10% FBS (Gibco), 0.1 mmol/L NEAA (Gibco), 1% penicillin/streptomycin (Gibco), 10 ng/mL bFGF (JPC) and 5 ng/mL TGF-β (PeproTech). After about 10 days, confluent fibroblast-like cells were collected and then seeded onto Gelatin-coated plates and cultured in hMSC culture medium. CD73/CD90/CD105 triple-positive cells were sorted by flow cytometer (BD, Aria II).

Generation of hVSMCs

The differentiation of *ALKBH1*$^{+/+}$ and *ALKBH1*$^{-/-}$ hVSMCs was performed as described previously

(Ling et al., 2019; Yan et al., 2019). In brief, hESCs were dissociated into single cells with TrypLE Express (Gibco). 3×10^5 hESCs were seeded onto Matrigel-coated 6-well plates (Corning) in mTeSR medium with the addition of ROCK inhibitor Y-27632 (Selleck). After one day, the medium was replaced with hVSMC medium M1 containing 50% DMEM/F12 (Gibco), 50% Neurobasal medium (Gibco), 1 × B27 supplement without Vitamin A (Gibco), 1 × N2 supplement (Gibco), 1% penicillin/streptomycin (Gibco), 55 μM β-Mercaptoethanol (Thermo Fisher Scientific), 7 μM CHIR99021 (Selleck) and 25 ng/mL BMP4 (R&D) for 3 days. The medium was then replaced with hVSMC medium M2 containing 50% DMEM/F12 (Gibco), 50% Neurobasal medium (Gibco), 1 × B27 supplement without Vitamin A (Gibco), 1 × N2 supplement (Gibco), 1% penicillin/streptomycin (Gibco), 55 μM β-Mercaptoethanol (Thermo Fisher Scientific), 2 ng/mL Activin A (StemImmune) and 10 ng/mL recombinant Human PDGF-AB (PeproTech) for 2 days. CD140b-positive cells were sorted by flow cytometer (BD, Aria II).

Fluorescence-activated cell sorting (FACS)

hESCs, hMSCs and hVSMCs were dissociated into single cells with TrypLE Express (Gibco). Cells were washed twice with PBS. Cells were incubated in antibodies diluted with 10% FBS in PBS for 1 hr at room temperature. Positive cells were sorted by flow cytometer (BD, Aria II). Antibodies used for FACS are anti-CD73 (550741, BD Biosciences), anti-CD90 (555595, BD Biosciences), anti-CD105 (17-1057, eBioscience) and anti-CD140b (558821, BD Biosciences).

Clonal expansion assay

Single-cell clonal expansion assay was performed as described previously (Cheng et al., 2019; Deng et al., 2019). In brief, 2,000 hMSCs were seeded onto Gelatin-coated 12-well plates (Corning), cultured for approximately 10 days until nearly confluent, fixed with 4% paraformaldehyde for 30 min at room temperature, and then stained with crystal violet for 30 min at room temperature. The relative cell density was quantified by ImageJ.

SA-β-Gal staining

SA-β-Gal staining was performed as described previously (Cheng et al., 2019; Deng et al., 2019). In brief, cells were washed by PBS and fixed by fixation fluid (2% formaldehyde and 0.2% glutaraldehyde in PBS) for 5 min at room temperature and then stained by SA-β-Gal staining solution overnight at 37 °C. The percentages of SA-β-Gal-positive cells were quantified by ImageJ.

Wound healing assay

6×10^4 hVSMCs were seeded onto Gelatin-coated 96-well plates (Corning). At a confluence of 90%, hVSMCs were scratched and photographed by IncuCyte S3 (ESSEN BIOSCIENCE) for 24 hr. The cell migration capacity was then analyzed by ImageJ.

Measurement of cell apoptosis

Cells were collected freshly with TrypLE Express (Gibco), stained with Annexin V-EGFP Apoptosis Detection Kit (Vigorous Biotechnology) following the manufacturer's instructions and analyzed with a flow cytometer (BD FACS Calibur).

Western blotting

1×10^6 cells were lysed in 2 × SDS lysis buffer (100 mM Tris-HCl, pH 6.8, 20% Glycerol, 4% SDS and 4% β-Mercaptoethanol) at 105 °C for 5 min and quantified with a BCA quantification kit (Thermo Fisher Scientific). 20 μg of protein per sample was subjected to SDS-PAGE and electrotransferred to PVDF membranes (Millipore). The membranes were then blocked with 5% non-fat milk for 1 hr at room temperature, incubated with primary antibodies at 4 °C overnight and horseradish peroxidase (HRP)-conjugated secondary antibodies for 1 hr at room temperature. Immunoreactive bands were captured with a ChemiDoc XRS system (Bio-Rad) and analyzed with ImageJ. Primary antibodies used for western blotting are anti-ALKBH1 (Abcam, ab126596), anti-GAPDH (Santa Cruz, sc-25778) and anti-β-Tubulin (Immunoway, YM3030). HRP-conjugated secondary antibodies are goat-anti-mouse (ZSGB-BIO, ZB2305) and goat-anti-rabbit (ZSGB-BIO, ZB2301).

Immunofluorescence microscopy

For the detection of cell type-specific markers, cells seeded on microscope coverslips were fixed with 4% paraformaldehyde for 30 min, permeabilized with 0.4% Triton X-100 for 30 min, blocked with 10% donkey serum (Jackson ImmunoResearch Laboratories) for 1 hr at room temperature, incubated with primary antibodies at 4 °C overnight and then incubated with fluorescence-labeled secondary antibodies at room temperature for 1 hr. Hoechst 33342 (Invitrogen) was used to stain nuclear DNA. Images were taken with a Leica SP5 confocal microscope.

Primary antibodies used for immunostaining are anti-OCT4 (Santa Cruz Biotechnology, sc-5279), anti-SOX2 (R&D, MAB2018), anti-NANOG (Abcam, ab109250), anti-Calponin (Dako, M3556), anti-SM22α (Abcam, ab14106), anti-53BP1 (Bethyl Laboratories, A300-273A) and anti-γH2AX (Millipore, 05-636). Secondary antibodies used for immunostaining are Alexa Flour 488 donkey anti-mouse (Invitrogen, A21202), Alexa Flour 568 donkey anti-goat (Invitrogen, A11057), Alexa Flour 647 donkey anti-rabbit (Invitrogen, A31573) and Alexa Flour 568 donkey anti-rabbit (Invitrogen, A10042).

JC-10 mitochondrial membrane potential assay

Detection of mitochondrial membrane potential was performed with Cell Meter™ JC-10 Mitochondrial Membrane Potential Assay Kit (AAT Bioquest, 22801) following the manufacturer's instructions.

PCR detection of potential mycoplasma contaminations

Cell culture medium was used as the template for PCR to detect potential mycoplasma contaminations. PCR products were separated by electrophoresis on 1% agarose gel and bands were visualized with a ChemiDoc XRS system (Bio-Rad). Primers used for mycoplasma detection are shown as below.

Mycoplasma_Fw: 5′-GGGAGCAAACAGGATTAGATACCCT-3′;

Mycoplasma_Re: 5′-TGCACCATCTGTCACTCTGTTAACCTC-3′.

Isolation of genomic DNA

Genomic DNA (gDNA) was extracted with Blood/Cell/Tissue Genomic DNA Extraction Kit (TIANGEN). RNA was removed with RNase A treatment following the manufacturer's instructions

before DNA was eluted with nuclease-free water.

6mA dot blotting assay

6mA dot blotting assay was performed as previously reported (Wu et al., 2016). In brief, genomic DNA was denatured at 95 °C for 5 min and immediately transferred to ice and incubated for another 5 min. Samples were spotted on Immobilon-Ny^+ Membrane (Millipore), air dried for 5 min, and crosslinked with a UV-crosslinker (1,200 mJ/cm^2, 30 sec for three times; SPECTROLINKER). The membranes were then blocked with 5% non-fat milk in PBST (0.02% Tween-20 in PBS) for 1 hr at room temperature and incubated with anti-6mA antibody (SYSY, 202003) at 4 °C overnight and HRP-conjugated goat-anti-rabbit secondary antibody (ZSGB-BIO, ZB2301) for 1 hr at room temperature. Methylene blue staining was used as loading control. Immunoreactive dots were captured with a ChemiDoc XRS system and analyzed with ImageJ.

Quantification of 6mA in gDNA by LC-MS/MS

LC-MS/MS analysis was conducted as previously reported (Liu et al., 2016). In brief, 1 μg of genomic DNA was denatured at 100 °C for 5 min, cooled on ice for 5 min, digested by 1 U Nuclease P1 (Wako USA, 145-08221, Lot# CAJ3980) in 10 mM NH_4OAc (pH 5.3) at 42 °C overnight, and incubated at 37 °C for 2 hr with the addition of 3.4 μL NH_4HCO_3 (1 M) and 1 μL of phosphodiesterase I from crotalus adamanteus venom (0.001 U, Sigma, P3243-1VL, Lot# SLCC7135). Then, 1 U alkaline phosphatase from *E. coli* (Sigma, P5931-500UN, Lot# 126M40557V) was added and samples were incubated at 37 °C for another 2 hr, diluted with nuclease-free water and subjected to LC-MS/MS analysis for detection of 6mA. The nucleosides were separated by reverse phase ultra-performance liquid chromatography on a C18 column (Agilent, 927,700-092) with online mass spectrometry detection using a TSQ Triple Quadrupole Mass Spectrometers (Thermo Fisher Scientific) set to multiple reaction monitoring in the positive ion mode. The nucleosides were quantified by using the nucleoside-to-base ion mass transitions of m/z 266.1 to 150.0 (6mA) and m/z 252.1 to 136.1 (dA). Quantification was carried out by comparison with a standard curve obtained from pure nucleoside standards run with the same batch of samples. The ratio of 6mA to dA was calculated based on the calibrated concentrations.

Whole-genome sequencing and copy number variation analysis

For whole-genome sequencing, genomic DNA was extracted with Blood/Cell/Tissue Genomic DNA Extraction Kit (TIANGEN). Library preparation, quality control and sequencing were conducted by Novogene Bioinformatics Technology Co. Ltd. For data analysis, low-quality reads and Illumina adapters were trimmed using TrimGalore (v0.4.4_dev). Cleaned reads were mapped to the UCSC human hg19 genome using Bowtie2 (v2.2.9) (Langmead and Salzberg, 2012) with default parameters. Mapped reads from mitochondrial DNA and the Y chromosome and reads with low mapping quality (MAPQ score < 10) were filtered using Samtools (v1.9) (Li et al., 2009). Duplicated reads were also removed using Picard software (v1.113). Then we counted the number of reads in non-overlapping windows of 500 kb using readCounter function in hmmcopy_utils (https://github.com/shahcompbio/hmmcopy_utils). The gene copy number, GC content and mappability were corrected with HMMcopy Bioconductor R package (v1.26.0) (Ha et al., 2012). The manhattan plots were generated using qqman R package (v0.1.4).

Mapping of microbial genomes to hMSC whole-genome sequencing data

For the assessment of potential 6mA contaminations in the cell culture, five microbial species among the most common sources of contaminations, including *Mycoplasma genitalium* (G37), *Mycoplasma Hyorhinis* (SK76), *Staphylococcus aureus* (G37), *Escherichia coli* (NCTC8325) and *Aspergillus fumigatus* (Af293) were examined. The latest version of reference genomes for each species was downloaded from NCBI genome (https://www.ncbi.nlm.nih.gov/). Cleaned reads from the hMSC whole-genome sequencing data were mapped to these reference genomes using Bowtie2 (v2.2.9) (Langmead and Salzberg, 2012) with default parameters, with an overall alignment rate of 0 considered to be contamination-free.

RNA-seq

RNA-seq library preparation, sequencing and data processing were performed as previously described (Zhang et al., 2019). In brief, total RNA was extracted using TRIzol reagents from 1×10^6 cells per duplicate and genomic DNA was removed using a DNA-free kit (Thermo Fisher Scientific). Library construction, quality control and sequencing were conducted by Novogene Bioinformatics Technology Co. Ltd. For RNA-seq data processing, quality control of raw reads was performed using the FASTQC (v0.11.6). Low quality reads and Illumina adaptors were trimmed using TrimGalore (v0.4.4_dev) with default parameters. Trimmed reads were mapped to the UCSC human hg19 genome using HISAT2 software (v2.1.0) (Kim et al., 2015). Transcriptional expression level of each annotated gene was quantified using HTSeq (v0.6.1) (Anders et al., 2015). Differentially expressed genes (DEGs) were calculated using DESeq2 (v1.24.0) (Love et al., 2014) with a cutoff Benjamini-Hochberg adjust P value < 0.05 and absolute $\log_2$ (fold change) > 0.58. The Euclidian distance was calculated based on DESeq2 regularized-logarithm (rlog) normalized read count. To visualize the RNA-seq signal, we calculated the normalized read counts by RPKM (Reads Per Kilobase per Million mapped reads) for each 10-base pair (bp) bin using bamCoverage function in deepTools (v3.3.0) (Ramirez et al., 2016) software.

Statistical analysis

Two-tailed Student's *t*-test was conducted by using GraphPad Prism. Data are presented as the mean ± SEMs. P value < 0.05 is considered statistically significant. *, $P < 0.05$; **, $P < 0.01$; ***, $P < 0.001$.

Data availability

High-throughput sequencing data of this study have been deposited in Gene Expression Omnibus (GEO) database with accession number GSE150936.

Supplemental Figure Legends

Figure S1. Generation and characterization of *ALKBH1*$^{-/-}$ hESCs and hMSCs.

(A) Schematic diagram showing the outcome of CRISPR/Cas9 cleavage at exon 1. The Cas9-mediated DNA cleavage at exon 1 resulted in a 13-bp deletion on one allele and a 10-bp deletion on the other allele, both of which introduced a premature stop codon ("TGA" in red rectangle) at exon 2.

(B) Western blotting analysis of ALKBH1 in *ALKBH1*$^{+/+}$ and *ALKBH1*$^{-/-}$ hMSCs. GAPDH was used as a loading control.
(C) Whole genome analysis of copy number variations in *ALKBH1*$^{+/+}$ and *ALKBH1*$^{-/-}$ hMSCs.
(D) Heatmap showing the Euclidean distance to reflect the reproducibility of RNA-seq data in *ALKBH1*$^{+/+}$ and *ALKBH1*$^{-/-}$ hMSCs. The color key of the Euclidean distance from dark to light indicates strong to weak correlation, respectively.
(E) Immunostaining of DNA damage markers γH2AX and 53BP1 in *ALKBH1*$^{+/+}$ and *ALKBH1*$^{-/-}$ hMSCs at passage 3. Scale bar, 10 μm. Data are presented as the mean ± SEMs. $n = 3$. ns, not significant.
(F) Independently repeated quantification of 6mA levels in gDNA by LC-MS/MS in *ALKBH1*$^{+/+}$ and *ALKBH1*$^{-/-}$ hMSCs.
(G) Measurement of mycoplasma DNA by PCR showing the absence of potential mycoplasma contamination in the culture medium of *ALKBH1*$^{+/+}$ and *ALKBH1*$^{-/-}$ hESCs, hMSCs and hVSMCs. Black arrow indicates expected position of the positive band.

References

Anders, S., Pyl, P.T., and Huber, W. (2015). HTSeq--a Python framework to work with high-throughput sequencing data. Bioinformatics 31, 166-169.

Cheng, F., Wang, S., Song, M., Liu, Z., Liu, P., Wang, L., Wang, Y., Zhao, Q., Yan, K., Chan, P.*, et al.* (2019). DJ-1 is dispensable for human stem cell homeostasis. Protein Cell 10, 846-853.

Deng, L., Ren, R., Liu, Z., Song, M., Li, J., Wu, Z., Ren, X., Fu, L., Li, W., Zhang, W.*, et al.* (2019). Stabilizing heterochromatin by DGCR8 alleviates senescence and osteoarthritis. Nat Commun 10, 3329.

Ha, G., Roth, A., Lai, D., Bashashati, A., Ding, J., Goya, R., Giuliany, R., Rosner, J., Oloumi, A., Shumansky, K.*, et al.* (2012). Integrative analysis of genome-wide loss of heterozygosity and monoallelic expression at nucleotide resolution reveals disrupted pathways in triple-negative breast cancer. Genome Res 22, 1995-2007.

Kim, D., Langmead, B., and Salzberg, S.L. (2015). HISAT: a fast spliced aligner with low memory requirements. Nat Methods 12, 357-360.

Langmead, B., and Salzberg, S.L. (2012). Fast gapped-read alignment with Bowtie 2. Nat Methods 9, 357-359.

Li, H., Handsaker, B., Wysoker, A., Fennell, T., Ruan, J., Homer, N., Marth, G., Abecasis, G., and Durbin, R. (2009). The Sequence Alignment/Map format and SAMtools. Bioinformatics 25, 2078-2079.

Ling, C., Liu, Z., Song, M., Zhang, W., Wang, S., Liu, X., Ma, S., Sun, S., Fu, L., Chu, Q.*, et al.* (2019). Modeling CADASIL vascular pathologies with patient-derived induced pluripotent stem cells. Protein Cell 10, 249-271.

Liu, J., Zhu, Y., Luo, G.Z., Wang, X., Yue, Y., Wang, X., Zong, X., Chen, K., Yin, H., Fu, Y.*, et al.* (2016). Abundant DNA 6mA methylation during early embryogenesis of zebrafish and pig. Nat Commun 7, 13052.

Love, M.I., Huber, W., and Anders, S. (2014). Moderated estimation of fold change and dispersion for RNA-seq data with DESeq2. Genome Biol 15, 550.

Ramirez, F., Ryan, D.P., Gruning, B., Bhardwaj, V., Kilpert, F., Richter, A.S., Heyne, S., Dundar, F., and Manke, T. (2016). deepTools2: a next generation web server for deep-sequencing data analysis. Nucleic Acids Res 44, W160-165.

Wu, T.P., Wang, T., Seetin, M.G., Lai, Y., Zhu, S., Lin, K., Liu, Y., Byrum, S.D., Mackintosh, S.G., Zhong, M., *et al.* (2016). DNA methylation on N(6)-adenine in mammalian embryonic stem cells. Nature 532, 329-333.

Yan, P., Li, Q., Wang, L., Lu, P., Suzuki, K., Liu, Z., Lei, J., Li, W., He, X., Wang, S., *et al.* (2019). FOXO3-Engineered Human ESC-Derived Vascular Cells Promote Vascular Protection and Regeneration. Cell Stem Cell 24, 447-461 e448.

Zhang, X., Liu, Z., Liu, X., Wang, S., Zhang, Y., He, X., Sun, S., Ma, S., Shyh-Chang, N., Liu, F., *et al.* (2019). Telomere-dependent and telomere-independent roles of RAP1 in regulating human stem cell homeostasis. Protein Cell 10, 649-667.

Figure S1

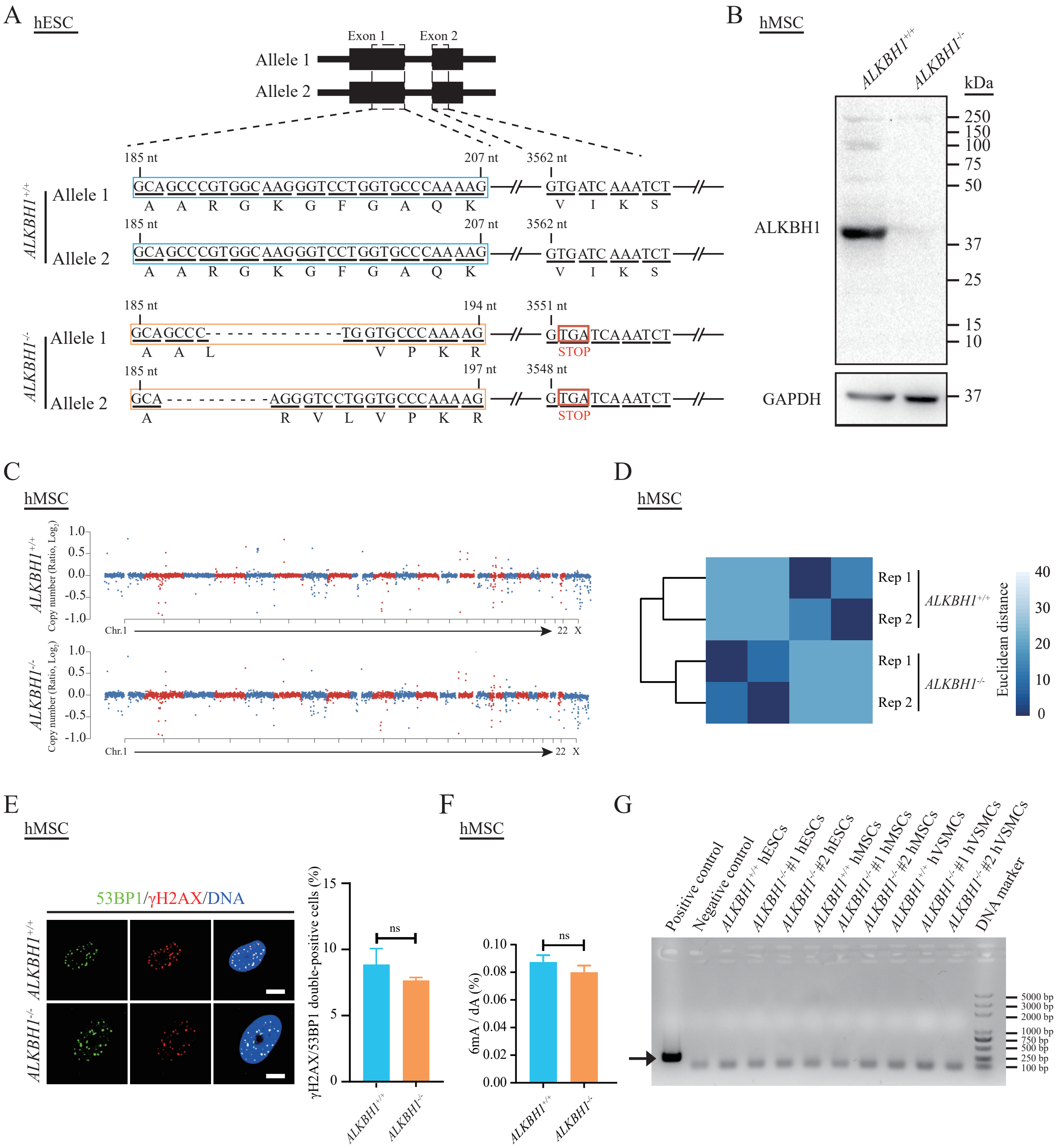
A
hESC
Exon 1
Exon 2
Allele 1
Allele 2
ALKBH1+/+
ALKBH1-/-
185 nt
207 nt
3562 nt
194 nt
3551 nt
197 nt
3548 nt
STOP
B
hMSC
ALKBH1+/+
ALKBH1-/-
kDa
250
150
100
75
50
37
25
15
10
ALKBH1
GAPDH
C
hMSC
Copy number (Ratio, Log2)
Chr.1
22
X
D
hMSC
Rep 1
Rep 2
Euclidean distance
40
30
20
10
0
E
hMSC
53BP1/γH2AX/DNA
γH2AX/53BP1 double-positive cells (%)
ns
F
hMSC
6mA / dA (%)
G
Positive control
Negative control
ALKBH1+/+ hESCs
ALKBH1-/- #1 hESCs
ALKBH1-/- #2 hESCs
ALKBH1+/+ hMSCs
ALKBH1-/- #1 hMSCs
ALKBH1-/- #2 hMSCs
ALKBH1+/+ hVSMCs
ALKBH1-/- #1 hVSMCs
ALKBH1-/- #2 hVSMCs
DNA marker
5000 bp
3000 bp
2000 bp
1000 bp
750 bp
500 bp
250 bp
100 bp
